# Interpopulation Variation in Contour Feather Structure Is Environmentally Determined in Great Tits

**DOI:** 10.1371/journal.pone.0024942

**Published:** 2011-09-19

**Authors:** Juli Broggi, Anna Gamero, Esa Hohtola, Markku Orell, Jan-Åke Nilsson

**Affiliations:** 1 Department of Biology, University of Oulu, Oulu, Finland; 2 Departamento de Humedales, Estación Biológica Doñana, CSIC, Sevilla, Spain; 3 Department of Sociobiology and Anthropology, University of Göttingen, Göttingen, Germany; 4 Department of Ecology, Animal Ecology, University of Lund, Lund, Sweden; University of Western Ontario, Canada

## Abstract

**Background:**

The plumage of birds is important for flying, insulation and social communication. Contour feathers cover most of the avian body and among other functions they provide a critical insulation layer against heat loss. Feather structure and composition are known to vary among individuals, which in turn determines variation in the insulation properties of the feather. However, the extent and the proximate mechanisms underlying this variation remain unexplored.

**Methodology/Principal Findings:**

We analyzed contour feather structure from two different great tit populations adapted to different winter regimes, one northern population in Oulu (Finland) and one southern population in Lund (Sweden). Great tits from the two populations differed significantly in feather structure. Birds from the northern population had a denser plumage but consisting of shorter feathers with a smaller proportion containing plumulaceous barbs, compared with conspecifics from the southern population. However, differences disappeared when birds originating from the two populations were raised and moulted in identical conditions in a common-garden experiment located in Oulu, under *ad libitum* nutritional conditions. All birds raised in the aviaries, including adult foster parents moulting in the same captive conditions, developed a similar feather structure. These feathers were different from that of wild birds in Oulu but similar to wild birds in Lund, the latter moulting in more benign conditions than those of Oulu.

**Conclusions/Significance:**

Wild populations exposed to different conditions develop contour feather differences either due to plastic responses or constraints. Environmental conditions, such as nutrient availability during feather growth play a crucial role in determining such differences in plumage structure among populations.

## Introduction

Plumage is the most diagnostic trait in birds and plays an essential role in flying, insulation and social communication. As plumage deteriorates, each individual periodically sheds and grows a variety of feathers with different structures and functions. Variation in feather quality has been widely studied, as it is well known that environmental and physiological conditions affect their structure, and therefore it can be used as an indicator of body condition [1]–[3]. Most studies have focused on the variation in growth bars of flight (pennaceous) feathers as these are crucial in flight performance (e.g. [4]), and easily identified and measured [5]. Since production of feathers is costly in terms of time, energy and nutrients, an individual producing a high-quality plumage may have to trade-off such costs against other costly processes like feather growth rate [6], reproductive effort [7]–[10] or migration [11], [12].

Contour feathers cover most of the avian body providing insulation from the environment. They are composed of a shaft with regularly spaced branches (barbs) on each side, which are in turn equally branched with barbules. The number of barbs and barbules, and the way they are attached to each other largely determines how much air they can trap, and thus the insulating properties of the feathers [13]. Furthermore, contour feathers also play a key role in social communication as the number, position and growth dynamics of barbs and barbules also influence the deposition of pigments and the ultrastructure of the feather. Such properties ultimately determine the feather's visual characteristics, and thereby their signaling properties [14], [15]. Despite knowledge on how differences in feather structure originate [16], information on how feather composition and structure vary between individuals or populations is scant.

Birds lose heat mostly by conduction and convection to the surroundings as long as they maintain a bodily surface temperature that is higher than the ambient temperature [17], [18]. Feather structure, quality and quantity are crucial in regulating such heat transfer processes, providing a critical buffer against this thermal gradient [18], [19]. Plumage characteristics are defined and fixed at the time of moulting, and subsequent modulations of the plumage insulation capacity are limited (but see [20]). Thus the number and structure of feathers sets an upper limit to insulation capacity. Some studies have found that within species, populations differing in winter conditions vary in their thermal conductance [21]. Likewise, the mass of contour feathers has been found to vary between populations from different origins, and also within populations as part of a seasonal acclimatization process [22]–[27]. Furthermore, it has been suggested that reductions in thermal conductance would not only depend on increased number of feathers, but probably also on changes in feather structure [22], [24], [28], [29].

However, as moult is costly [30], [31], energy and time constraints may preclude the production of a plumage that is optimal with respect to insulation properties. Moult rate has been shown to affect plumage structure, which in turn may affect future survival and reproductive performance [32], [6]. Furthermore, several studies have shown that investment in moult may be traded–off against reproductive effort [7]–[10], especially in northern populations were these activities may overlap extensively [33]. Hence, both the time for moult and the resource availability during moult probably decreases with latitude, potentially constraining individuals from northern populations to grow an optimal plumage.

The great tit (*Parus major L.*) is a year-round resident passerine species widespread over Europe. Populations across such an extensive latitudinal gradient are faced with marked seasonal differences in conditions such as food availability and climate. Thus, great tit populations in northern Europe experience shorter and delayed breeding seasons that in turn may shorten their moulting period compared with their southern counterparts [33]. This may result in the production of a plumage with reduced insulating quality [32]. On the other hand, as northern populations are faced with harsher winters, plumage structure potentially plays a more important role and should thereby be predicted to be of higher insulating quality when compared with southern populations.

In previous studies, we have shown that great tits from Oulu, northern Finland (north population) and Lund, southern Sweden (south population), are locally adapted to respond metabolically to the prevailing environmental conditions [34], [35]. However, whether plumage characteristics differ between populations and to what extent feather structure is intrinsically determined remains unknown. We studied the structure of the contour feathers of great tits in order to find out whether there are differences between birds from these two populations. We further employed a common-garden design in order to reveal whether there is a genetic component in the expected differences in contour-feather structure or if these are due to a plastic response to the local conditions such as nutrient availability.

## Results

**Figure 1:**
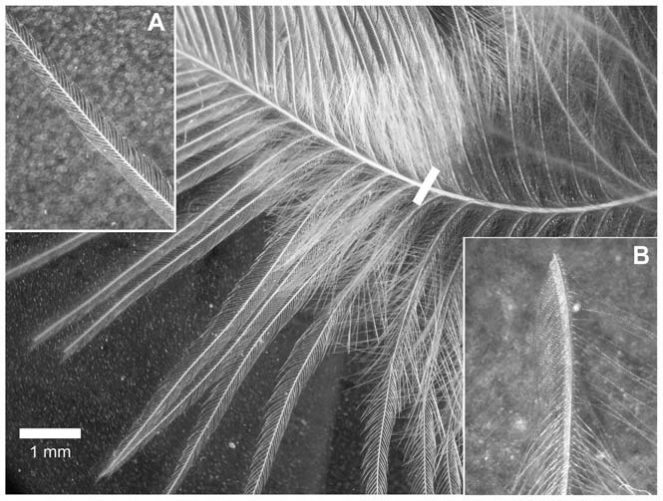
Structure of a great tit contour feather. Illustration of the different parts of a contour feather from the sternal tract of a great tit. The pennaceous barbs on the upper portion and plumulaceous barbs on the lower portion of the rachis are delimited by a white stripe. Details of the pennaceous (A) and plumulaceous barbs (B) with their corresponding barbules are shown as insets.

We summarized feather structure by the first factor of a principal component analysis including all the feather variables measured, the rest of the factors having eigenvalues <1 (see Figure 1). Density of both types of barbs and barbules (pennaceous and plumulaceous) were positively related and varied accordingly, opposite to feather length and the relative proportion of the two types of barbs (Table 1). Overall, birds with a high value of the first factor had feathers which were denser but shorter and with a lower proportion of plumulaceous barbs.

**Table 1:** Interrelation among descriptive contour feather variables.

	Factor 1
Eigenvalue	3.97
Variance explained	66.1
Variables	
Total_Length	−0.866
Density of Pl. barbules	0.776
Density of Pn. barbules	0.884
Density of Pl. Barbs	0.764
Density of Pn. Barbs	0.855
Proportion of Pl. Barbs	−0.720

Variables describing contour feather structure from the sternal tract of great tits. Total feather length (without calamus); densities of pennaceous (Pn) and plumulaceous (Pl) barbs and barbules; and proportion of each feather composed by plumulaceous barbs. Eigenvalue and coefficient of determination of the first factor obtained from a principal component analysis summarising overall feather structure, together with the factor loadings of each variable.

**Figure 2:**
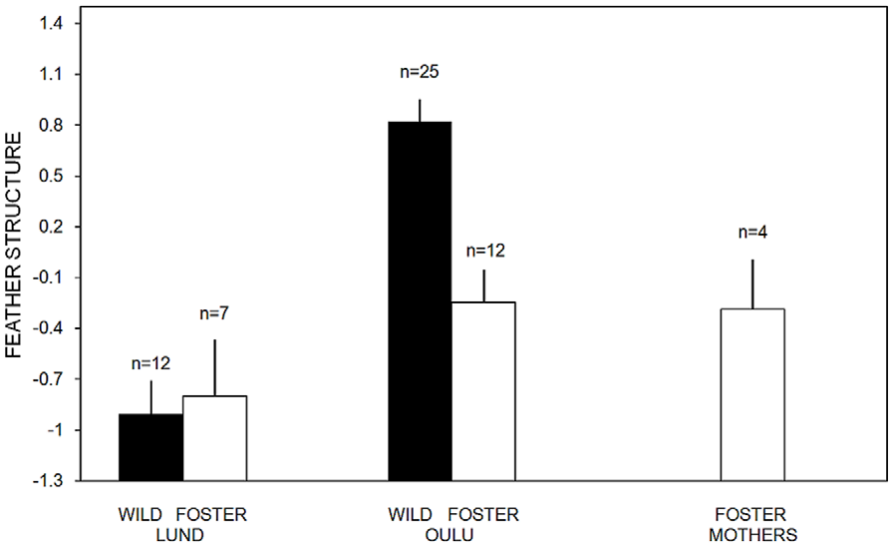
Variation in contour feather structure among wild and “common-garden” great tits. Differences in structure of contour feathers as estimated from the first principal component of six feather variables (see Table 1) from the sternal tract of wild (black bars), and foster (white bars) great tits originating from Lund and Oulu, with the corresponding error bars. Foster parents from Oulu (white bars) spent the same time as foster juveniles inside the aviaries.

Wild birds from Oulu had a denser plumage that consisted of shorter feathers with a smaller proportion of plumulaceous barbs than wild birds from Lund (Table 2). This was corroborated by a significant difference in feather structure, as measured by the first factor of the principal component analysis among the individuals from the two populations (ANOVA: F_1,35_ = 52.7; P<0.001; R^2^ = 0.60; Figure 2). Neither age (F_1,33_ = 0.109; P = 0.74) nor sex (F_1,33_ = 0.008; P = 0.93) or their interactions with population of origin (P>0.1) accounted for any significant variation in feather structure.

**Table 2:** Descriptive variables for the contour feathers.

	Lund Wild	Oulu Foster	Oulu Wild
Density of pennaceous barbs (per mm)	1.32±0.18^a^	1.40±0.16^a^	1.61±0.21^b^
Density of plumulaceous barbs (per mm)	2.71±0.31^a^	3.01±0.20^b^	3.15±0.33^b^
Density of pennaceous barbules (per 0.1 mm)	1.90±0.13^a^	2.10±0.17^b^	2.32±0.16^c^
Density of plumulaceous barbules (per 0.1 mm)	2.52±0.23^a^	2.58±0.22^a^	2.89±0.21^b^
Proportion of plumulaceous barbs (%)	73.5±4.0^a^	74.7±3.4^a^	70.6±2.6^b^
Length of the feather (mm)	24.2±1.65^a^	21.2±2.46^b^	19.1±2.15^c^

Mean ± SD density of four feather variables, proportion of plumulaceous barbs and length of feathers among wild caught birds from Lund (N = 12) and from Oulu (N = 25) as well as foster juveniles originating from Oulu but moulting in aviaries (N = 12). Different superscript letters denote statistically significant (P<0.05) differences as determined from ANOVAs with Tukey post-hoc tests.

However, birds originating from the two populations, but raised in Oulu under identical conditions did not differ in their feather structure (F_1,18_ = 2.33; P = 0.145; R^2^ = 0.12; Figure 2). Further, the four foster parents that moulted in an identical aviary setting, had a similar feather structure as the foster juveniles (Tukey post-hoc test: P = 0.99) but a significantly lower estimate of the first principal component compared to wild birds from Oulu (Tukey post-hoc test: P = 0.011; Figure 2). When all wild and foster birds (except foster parents) were analyzed together, wild birds from Oulu had on average a higher score of the first principal factor than wild birds from Lund or foster birds (Table 3; Figure 2). Further, the interaction between population and manipulation (wild vs. foster) was highly significant due to a marked decrease in the first principal factor for foster birds compared to wild birds in Oulu which was not evident for Lund birds (Table 3; Figure 2). Additionally, when looking at the different feather variables separately, foster juveniles from Oulu had a significantly less dense plumage except for density of plumulaceous barbs, a significantly higher proportion of plumulaceous barbs, and longer feathers than wild Oulu birds (Table 2).

**Table 3:** The results of an ANOVA explaining the variation in feather structure.

	df	F	P
Population	1	29.9	<0.001
Manipulation	1	5.36	0.025
Population x Manipulation	1	7.94	0.007
Error	52		

Significant explanatory variables of the variation in the first factor obtained from a principal component analysis (Table 1). Population denotes great tits from Lund or Oulu and Manipulation denotes wild or foster birds. R^2^ = 0.55.

In sum all birds that moulted inside the aviaries in Oulu, with *ad libitum* access to food, developed similar kind of feathers, approaching the values of wild birds from Lund. The feathers grown by wild Oulu birds differed consistently from all the others in being shorter, denser, and with a lower proportion of plumulaceous barbs (Figure 2).

## Discussion

Contour feather structure varied between the two wild populations. Great tits from the northern population developed denser but shorter feathers, and with a lower proportion of plumulaceous barbs (Figure 1) compared to conspecifics from the southern population. Such differences may be interpreted as local adaptations to wintering conditions, although it is hard to predict which of the two feather structures would provide the best thermal insulation. If a denser plumage is more important than the proportion of plumulaceus barbs or the length of the feathers, northern birds would have better insulation than those at southern locations. So far, the few studies exploring differences in feather structure among individuals have focused on seasonal differences among individuals. As a general trend, plumage weight was found to increase together with insulation capacity in winter acclimatized birds as compared to individuals during summer. These changes were mostly ascribed to variation in the number of feathers [36], [27], but see [24], [29]. In the only study analyzing feather structure in relation to environmental conditions, winter acclimatized American goldfinches (*Carduelis tristis*) developed a plumage with denser feathers and with a higher proportion of plumulaceous barbules [24]. Although the present study focuses on interpopulation differences rather than seasonal, the supposedly optimal combination of traits (denser and more plumulaceous feathers) was not found in the studied great tit populations.

However, the differences in feather structure disappeared when birds from the two populations moulted inside the Oulu aviaries, with *ad libitum* food in common-garden conditions (Figure 2). Likewise, adult birds from Oulu that acted as foster parents, and experienced the same conditions as their foster chicks, also developed feathers of the same structure as those developed by wild birds from Lund. Thus, feather structure seems to be a plastic response to energy and nutrient availability with a small scope for heritable variation as it has recently been suggested for growth rate of feathers [37]. Furthermore, all individual feather variables changed in consort. All of the foster juvenile estimates approached those from wild Lund birds, indicating that such characters are phenotypically integrated (Table 2). Thus, there seems to be a negative relation between the density of barbs and barbules on one hand, and the length as well as the proportion of the feather consisting of plumulaceous barbs on the other. The constraints responsible for this trade-off between feather characteristics are intriguing but a more definite answer has to await further experimentation.

Our results from the common garden experiment indicate that the difference between the wild birds from Lund and Oulu depends on time and energy constraints during the moulting period. The benign conditions encountered in the aviaries at the time of moult coincides with a period when wild birds experience low energy availability compared to the need for growing feathers of maximum quality [32], [1]. The constraints of energy and nutrient availability on feather production may be especially severe in resident birds at high latitudes as breeding terminates later in relation to the onset of winter, and females produce too large clutches in relation to food availability [38] than birds at lower latitudes. Thus, our results suggest that feathers grown by wild birds in Lund i.e. long feathers with a large proportion of plumulaceous barbs, would have better insulation properties than the feathers grown by wild birds from Oulu. Furthermore, this interpretation is in line with a previously unexplained result from an earlier study in the two populations, in which great tits from Oulu expended more energy on thermoregulation at −10°C than did Lund birds [34]. Altogether, these results suggest that birds from Oulu are unable to produce optimal feathers due to time and/or nutrient constraints, which may result in the development of a plumage with poorer insulation properties as compared to their conspecifics from Lund. Further, this may partly explain the low winter survival experienced at northern latitudes such as in Oulu, as such populations seem to persist due to the influx of immigrants from the south (Karvonen et al. in prep.).

Nevertheless, as our measured feather characters varied in parallel we cannot exclude that some other co-varying character, like the total number of feathers or variation in other feather tracts may be relevant for the overall insulating properties of the plumage. Certainly, more studies are required to understand what factors trigger the development of different feather structures among populations of the same species, and the fitness consequences of such differences.

## Materials and Methods

### Ethics Statement

All procedures were approved by the ethical committee of the University of Oulu, #097/04.

From January to March 2001, 12 great tits from Lund (55°40′N, 13°25′E) and 25 from Oulu (65°N, 25°30′E) were captured and five contour feathers from the side of the breast, the ventral-sternal tract (between the shoulder and the breast black stripe), were plucked from each individual. Feathers were plucked and handled with tweezers, and otherwise stored in dry paper envelopes. Details on the respective study areas and capturing procedure are provided elsewhere [34].

### Common garden experiment

Thirty great tit eggs from Lund (55°40′N, 13°25′E) were removed from different nests (two eggs per nest) soon after lying and stored at +4°C and then brought to Oulu by plane. Another 30 eggs were gathered from nests in the study area in Oulu (65°N, 25°30′E).

Two days after incubation started, we replaced the original clutches of wild great tit nests located within the Oulu study area by foster eggs. Thus, foster parents incubated homogeneous clutches from either Oulu or Lund. Just before fledging, nestboxes with the chicks were moved inside aviaries together with one of the parents. Parents continued to feed the chicks during the first weeks after fledging inside the aviaries, and after a few weeks chicks were able to feed for themselves. At six weeks of age, fledglings were individually ringed, separated from their foster parents and reallocated to the aviaries so that each individual shared a cage with no more than six other birds from the same origin. Roosting nestboxes and feeders with *ad libitum* food were also installed according to the number of birds per cage. Diet consisted of vitamin-enriched mixture of diverse seeds, pork fat and live protein to ensure birds were properly nourished. Details on the precise methodology to maintain the caged birds are provided elsewhere [35]. Feathers from all birds were plucked according to the same methodology as described above during two weeks from late October to early November 2003 when both foster young and parents had completed their moult.

### Feather structure

Feathers were investigated with the help of a stereoscopic microscope with an ocular grid. To describe feather structure we measured 6 different traits [24]. For each feather, the division between the pennaceous (Figure 1A) and plumulaceous (Figure 1B) sections of the feather was determined and both types of barbs counted (20×); the total length of the feather without calamus and the length of the plumulaceous barb zone were measured at 6×(see [13] for a detailed description of feather structural components). The number of barbules from both plumulaceous and pennaceous barbs was counted from a fixed portion (0.43 mm) of barb, starting 0.43 mm from the rachis at 70×(see Figure 1 for detailed illustration). Thus, the variables measured were the density of barbs and barbules from the plumulaceous and pennaceous portions of the feather, the proportion of plumulaceous barbs with respect to all barbs, and the total feather length (excluding calamus). These variables were estimated on two feathers per individual in order to obtain repeatability estimates. Repeatability was significant for all feather variables as judged by a one-way ANOVA with individual as grouping factor (r = 0.38–0.71; all P<0.001). Average values of the two measurements were used for later analyses. Due to the level of intercorrelation among variables, we used the first factor of a principal component analysis (using a correlation matrix and without factor rotation) to describe feather structure. All the feather measurements were done by the same person (A.G.).

All variables fulfilled the requirements of normality (tested with the Kolmogorov–Smirnov and Shapiro-Wilk one sample test) and thus parametric statistics were used in all analysis.
